# Control of clustered action potential firing in a mathematical model of entorhinal cortex stellate cells

**DOI:** 10.1016/j.jtbi.2018.04.013

**Published:** 2018-07-14

**Authors:** Luke Tait, Kyle Wedgwood, Krasimira Tsaneva-Atanasova, Jon T. Brown, Marc Goodfellow

**Affiliations:** a Living Systems Institute, University of Exeter, Exeter, United Kingdom; b College of Engineering, Maths, and Physical Sciences, University of Exeter, Exeter, United Kingdom; c Centre for Biomedical Modelling and Analysis, University of Exeter, Exeter, United Kingdom; d EPSRC Centre for Predictive Modelling in Healthcare, University of Exeter, Exeter, United Kingdom; e University of Exeter Medical School, Exeter, United Kingdom

**Keywords:** Dementia, Bifurcation analysis, Neuron model, Bursting, Subthreshold oscillations

## Abstract

The entorhinal cortex is a crucial component of our memory and spatial navigation systems and is one of the first areas to be affected in dementias featuring tau pathology, such as Alzheimer’s disease and frontotemporal dementia. Electrophysiological recordings from principle cells of medial entorhinal cortex (layer II stellate cells, mEC-SCs) demonstrate a number of key identifying properties including subthreshold oscillations in the theta (4–12 Hz) range and clustered action potential firing. These single cell properties are correlated with network activity such as grid firing and coupling between theta and gamma rhythms, suggesting they are important for spatial memory. As such, experimental models of dementia have revealed disruption of organised dorsoventral gradients in clustered action potential firing.

To better understand the mechanisms underpinning these different dynamics, we study a conductance based model of mEC-SCs. We demonstrate that the model, driven by extrinsic noise, can capture quantitative differences in clustered action potential firing patterns recorded from experimental models of tau pathology and healthy animals. The differential equation formulation of our model allows us to perform numerical bifurcation analyses in order to uncover the dynamic mechanisms underlying these patterns. We show that clustered dynamics can be understood as subcritical Hopf/homoclinic bursting in a fast-slow system where the slow sub-system is governed by activation of the persistent sodium current and inactivation of the slow A-type potassium current. In the full system, we demonstrate that clustered firing arises via flip bifurcations as conductance parameters are varied. Our model analyses confirm the experimentally suggested hypothesis that the breakdown of clustered dynamics in disease occurs via increases in AHP conductance.

## Highlights

• An SDE model of entorhinal cortex (EC) stellate cells is proposed.
• Experimentally observed action potential clustering is investigated in the model.
• Clusters are generated by subcritical-Hopf/homoclinic type bursting.
• Potential mechanisms underlying changes in EC dynamics in dementia are presented.

## 1 Introduction

The entorhinal cortex occupies a key role in the cortical-hippocampal circuit, acting as a gateway between the neocortex and hippocampus (Canto et al., 2008) and playing a pivotal role in working memory processing and spatial navigation (McNaughton, Battaglia, Jensen, Moser, Moser, 2006, Moser, Kropff, Moser, 2008). Many different functional cell types involved in the coding of spatial representation are found in the entorhinal cortex, including grid cells, border cells, head direction cells and speed cells (Giocomo, Stensola, Bonnevie, Cauter, Moser, Moser, 2014, Hafting, Fyhn, Molden, Moser, Moser, 2005, Kropff, Carmichael, Moser, Moser, 2015, Solstad, Boccara, Kropff, Moser, Moser, 2008). Spatial information from these cells is transferred from Layer II of the entorhinal cortex to place cells in the hippocampus, which in turn feed back into the entorhinal cortex (Barak, Feldman, Okun, 2015, Deng, Aimone, Gage, 2010, O’Keefe, Burgess, Donnett, Jeffery, Maguire, 1998).

The principle neurons in layer II of the medial entorhinal cortex are reported to be predominantly (60–70%) stellate cells (mEC-SCs) (Alonso, Klink, 1993, Booth, Ridler, Murray, Ward, de Groot, Goodfellow, Phillips, Randall, Brown, 2016). Analysis of recordings of mEC-SCs in brain slices demonstrates a number of key identifying electrophysiological properties, including a large membrane potential sag mediated by a hyperpolarisation activated cation current ($I_{\text{h}}$), subthreshold oscillations in the theta (4–12 Hz) range and clustered action potential firing (Alonso and Klink, 1993). Dorsoventral gradients in these electrophysiological properties (Booth, Ridler, Murray, Ward, de Groot, Goodfellow, Phillips, Randall, Brown, 2016, Dodson, Pastoll, Nolan, 2011, Garden, Dodson, O’Donnell, White, Nolan, 2008, Giocomo, Hasselmo, 2008, Giocomo, Hasselmo, 2009, Giocomo, Zilli, Fransen, Hasselmo, 2007) reflect similar dorsoventral gradients in grid cell spacing (Hafting et al., 2005), implying a key role in spatial memory.

The disruption of memory systems is one of the hallmarks of dementia (McGowan et al., 2006). The most common cause of dementia, Alzheimer’s disease, has been shown to affect the entorinhal cortex early in disease progression (Braak and Braak, 1991). One of the two primary pathologies of Alzheimer’s disease is the presence of neurofibrillary tangles caused by mutant forms of tau proteins (the other being plaques formed by amyloid beta). Experimental models of tau pathology have revealed that neurofibrillary tangles cause spatial memory deficits (Fu et al., 2017) that may be underpinned by alterations in the intrinsic cellular dynamics described above (Booth, Ridler, Murray, Ward, de Groot, Goodfellow, Phillips, Randall, Brown, 2016, Fu, Rodriguez, Herman, Emrani, Nahmani, Barrett, Figueroa, Goldberg, Hussaini, Duff, 2017). It is therefore crucial if we wish to develop treatments and therapries to build our understanding of the mechanisms underlying mEC-SC dynamics so that we can further elucidate the cellular and network bases of spatial memory, and ultimately the causes and consequences of Alzheimer’s disease.

There are many potential dynamical frameworks within which to mathematically model clustered firing of neurons or the generation of subthreshold oscillations. Phenomenological models have used extrinsic rhythmic inputs to drive integrate-and-fire type neurons across bifurcations (Pastoll, Solanka, Rossum, Nolan, 2013, Solanka, van Rossum, Nolan, 2015), thus producing temporal periods of quiescence interspersed with bursts of action potentials, that may be reminiscent of clustered firing. Low dimensional neuronal models such as the Izhikevic neuron (which is a non-linear integrate-and-fire type neuron) have been used to model mEC-SC firing patterns (Izhikevich, 2007, Shay, Ferrante, Chapman, Hasselmo, 2016) but are also constructed from a phenomenological, dynamical systems perspective and do not offer mechanistic insight at the single neuron level. For example, they do not allow understanding of the relationship between properties of membrane channels and the aforementioned dynamic firing patterns.

In order to develop a mechanistic, biophysical understanding, Fransén et al. (2004) developed a detailed, compartmental model of an mEC-SC, based on the Hodgkin–Huxley formulation. In addition to standard Hodgkin–Huxley ion channels, hyperpolarisation-activated, cation non-selective channels ($I_{\text{h}}$) were incorporated along with calcium-gated potassium channels including a potassium-mediated after-hyperpolarisation (AHP) current. It was demonstrated that this combination of channels was sufficient to describe limit cycle subthreshold oscillations in the theta (4–12 Hz) range and clustered action potential firing. A simulation study of the noise driven system demonstrated a dependence of clustered firing on the AHP conductance and the time scale of the slow $I_{\text{h}}$ component (Fransén et al., 2004). To investigate the role that stochastic effects could play in generating stellate cell dynamics, Dudman and Nolan (2009) formulated a high dimensional, Markov chain model of stochastic ion channel gating and demonstrated that this model could reproduce the aforementioned dynamics due to intrinsic ion channel noise. Clustered action potential firing was generated by a transient increase in probability of action potential firing during recovery from the AHP. This required the $I_{\text{h}}$ current, since simulations and experimental investigation of an $I_{\text{h}}$ knockout resulted in loss of clustering.

These previous models have provided insight into the potential biophysical mechanisms underpinning the clustered action potential firing and subthreshold oscillations of mEC-SC. However, the dynamic mechanisms underpinning clustered action potential firing were not elucidated, which precludes a thorough understanding of the ways in which changes in parameters affect dynamics. Such understanding would help to build a more complete picture of the reasons why different firing patterns can emerge, for example due to diseases such as Alzheimer’s disease. Furthermore, previous models have been cumbersome, either due to their dependence on calcium gated-channels or stochastic simulations. A simpler model would allow us to extend more readily into neuronal networks in the future in order to better understand the spatial structures underpinning memory processing in health and disease.

In order to advance such a framework, in this study, the model of Dudman and Nolan (2009) is converted to the deterministic Hodgkin-Huxley formulation. This results in an ordinary differential equation (ODE) model that retains the key components of $I_{\text{h}}$ and $I_{\text{AHP}}$. As a single compartment model with only voltage-gated ion channels, this model is simpler than the multi-compartment model of Fransén et al. (2004) which includes both voltage- and calcium-gated ion channels. Upon introducing extrinsic noise to the membrane potential in a stochastic differential equation (SDE) framework, numerical simulations are used to demonstrate that this model is capable of generating clustered action potential firing as well as subthreshold membrane potential fluctuations with peak power in the theta band, in line with experimental results. Numerical bifurcation analyses demonstrate that clustered firing in the model arises due to a flip bifurcation (Barrio, Shilnikov, 2011, Channell, Cymbalyuk, Shilnikov, 2007). Clustered action potential firing can, in turn, be understood in terms of a fast-slow system, in which the activation of the persistent sodium (NaP) and inactivation of the slow A-type potassium (Kas) channels act as slow variables, driving the fast sub-system through a hysteresis loop via subcritical Hopf and homoclinic bifurcations. Thus, in terms of the underlying dynamics, this model can be classified as a subcritical Hopf/homoclinic burster (Izhikevich, 2000). This model allows for clustered action potential firing to be controlled, making it a suitable model to study the role of dorsoventral gradients in clustering. It is thereby proposed that alterations to AHP or $I_{\text{h}}$ conductances could mediate the quantitative changes in clustering observed experimentally. In experimental models of dementia (rTg4510), loss of clustered firing is found to correlate with significant changes to AHP amplitude but no change in $I_{\text{h}}$ mediated sag (Booth et al., 2016). Hence our results suggest a possible path through parameter space that account for the differences in patterned firing in rTg4510.

## 2 Materials and methods

### 2.1 Mathematical model

The stochastically gated Markov Chain model of layer II medial entorhinal cortex stellate cells (mEC-SCs) presented by Dudman and Nolan (2009) was converted to a system of stochastic differential equations (SDEs) in the Hodgkin–Huxley formulation (Hodgkin and Huxley, 1952). For a given ion channel, Markov Chain models calculate the voltage dependent probability of a closed gate opening, *α*(*V*), and an open gate closing, *β*(*V*) in order to estimate the fraction of gates open at a given time. Under the assumption that the number of ion channels is sufficiently high, we can make a density approximation; i.e. the fraction of gates open is equal to the probability of gates being open, and hence we can write

(1) $$\frac{dx}{dt}=\alpha_{x}\left(V\right)\left(1-x\right)-\beta_{x}\left(V\right)x,$$

where *x* is the fraction of open gates for *x* in the set of ion channels. The presence of noisy fluctuations in the dynamics due to the intrinsic stochastic channel gating are not modelled explicitly, but approximated through the addition of extrinsic additive noise on the membrane potential.

The membrane potential is given by

(2) $$C\frac{dV}{dt}=I_{\text{app}}-I_{\text{NaT}}-I_{\text{NaP}}-I_{\text{Kdr}}-I_{\text{Kaf}}-I_{\text{Kas}}-I_{\text{h}}-I_{\text{AHP}}-I_{\text{L}}+\sigma \eta \left(t\right)$$

where the term *ση*(*t*) is the extrinsic noise term, where *σ* is the noise variance and 〈η(t)〉=0 and $\langle \eta \left(t\right),\eta \left(t^{\prime }\right)\rangle =\delta \left(t-t^{\prime }\right)$. Each ionic current is given by

(3) $$I_{X}=g_{X}\psi_{X}\left(V-E_{X}\right).$$

Here, *X* labels the set of ionic currents, *g_X_* is the maximal conductance of current *X, ψ_X_* is the fraction of channels in the conducting state (see Appendix A), and *E_X_* is the equilibrium potential of the current.

The transient sodium (NaT) and potassium delay rectifier (Kdr) are those of the classic Hodgkin–Huxley model and mediate action potential initiation and recovery respectively. Also included in the model are a persistent sodium (NaP) current, fast and slow potassium A-type currents (Kaf and Kas respectively), an Ohmic leak (L), and an inward hyperpolarisation activated (h) current.

Furthermore, a phenomenological spike-dependent outward after hyperpolarisation (AHP) current is included in the model. This current is modelled with $\alpha \left(V\right)=1.5\exp(-\left(t-t_{\text{spike}}\right)/\tau )$ and β=1.6. Here, *t*_spike_ is the time of the last spike (defined as membrane potential rising through 0 mV) and τ=60 ms such that the AHP lasts approximately 100 ms (Booth et al., 2016).

Noise variance was selected as follows. Having fixed all parameters but those being studied ($g_{\text{h}}$ and $g_{\text{AHP}}$), these remaining two free parameters of the deterministic system were chosen such that the inter-spike interval of the model reflected experimental results (Booth et al., 2016) ($g_{\text{h}}=2.8,$ $g_{\text{AHP}}=0.425$). The system was simulated for a range of noise values to identify plausible values with realistic clustering dynamics as quantified by *P_C_* (Nolan et al., 2007) (see Figs. S1 and S2, and description below). This yielded a value of $\sigma =0.197\;\mu A\cdot {\text{cm}}^{-2},$ or equivalently σ/C=0.135 mV · ms${}^{-1}$. This value was used in all stochastic simulations unless stated otherwise.

Simulations use the stochastic Heun method with a time step of 0.01 ms. Parameters are those given in Table 1 unless stated otherwise. For spectral analyses, the multitapered power spectrum was calculated using the CHRONUX toolbox (http://chronux.org/)(Mitra and Bokil, 2008) with 9 tapers and time-bandwidth product of 5.

A cluster of action potentials is defined as two or more spikes with an inter-spike interval of  < 250 ms, preceded and followed by a quiescent period of  > 300 ms. Clustering is quantified by *P_C_*, which is the ratio of spikes defined to be within a cluster to total number of spikes (Nolan et al., 2007). Calculation of *P_C_* is demonstrated in Fig. S1.

### 2.2 Bifurcation analysis

In order to understand the underlying dynamics, the ordinary differential equation (ODE) formalism is given by the above system with σ=0 in Eq. (2). This ODE formalism allows for a bifurcation analysis of the system. To conduct the bifurcation analysis, a number of methods were used. Equilibria were found using either XPPAUT (Ermentrout, 2002) or Matlab’s fsolve (MathWorks®, 2017) functions in a reduced system with no AHP current. This reduction is made since the AHP current is spike dependent and decays to zero in the absence of spikes.

Periodic orbits in the full model with AHP could not be analysed in XPPAUT due to the non-smooth nature of the AHP current. Instead, the Poincaré return map on the Poincaré section at V=0 (at which non-smoothness due to the AHP current arises) was identified using Matlab. For tonic spiking, high precision numerical solutions were found using a boundary value solver in Matlab. Due to the high dimensionality and complexity of the model, for doublets and other multiplets this could not be implemented. Instead solutions were found using Matlab’s ode45 (with tolerances set to $10^{-12}$) with high precision event detection, and the return map identified using Picard iterations; i.e. for each crossing of the Poincaré section, the Euclidian distance to all past crossing of the Poincaré section was calculated and a periodic orbit identified as this distance being less than $10^{-12}$. The Jacobian of the map was constructed by calculating Fréchet derivatives, and eigenvalues of the Jacobian used to assess stability and identify bifurcations in the map. Lyapunov exponents of the Poincaré return map were calculated to identify chaotic regimes (Sprott, 2003), where a negative maximum Lyapunov exponent (${\text{MLE}}_{\text{map}}$) represents a steady state on the map (corresponding to a stable limit cycle in the flow) and a positive ${\text{MLE}}_{\text{map}}$ represents a chaotic regime.

## 3 Results

### 3.1 Identifying parameter regimes of clustered firing

A number of experimental and modelling studies implicate the after hyperpolarisation (AHP) and hyperpolarisation activated current ($I_{\text{h}}$) in playing a role in clustered action potential firing (Booth, Ridler, Murray, Ward, de Groot, Goodfellow, Phillips, Randall, Brown, 2016, Dudman, Nolan, 2009, Fransén, Alonso, Dickson, Magistretti, Hasselmo, 2004, Nolan, Dudman, Dodson, Santoro, 2007). Motivated by these studies, the effect of the AHP and h-current conductances ($g_{\text{AHP}}$ and $g_{\text{h}}$ respectively) on clustering was studied in our model.

**Fig. 1:**
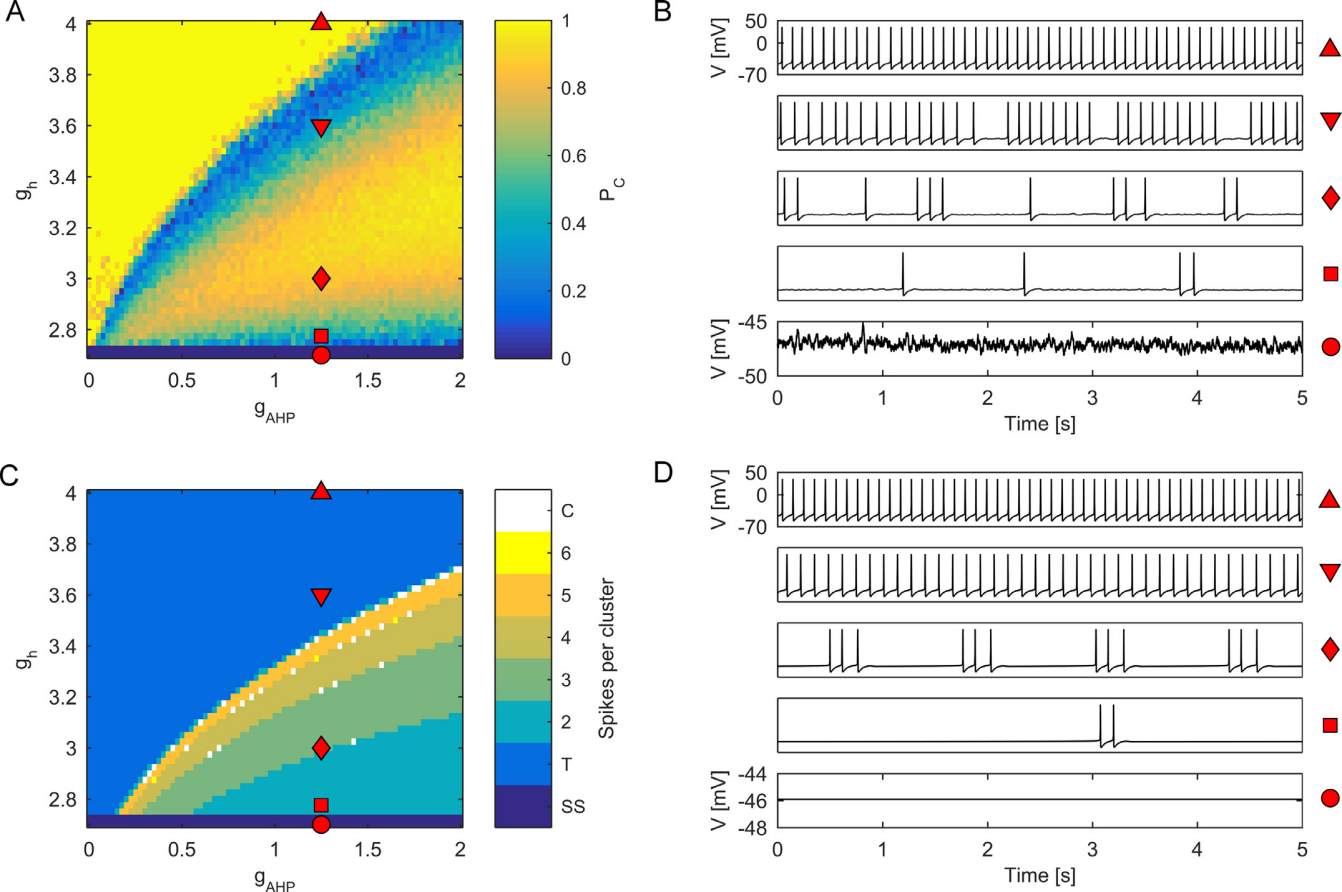
Clustered parameter regimes in two parameter space. (A) Heatmap of *P_C_* over a range of values of $g_{\text{AHP}}$ and $g_{\text{h}}$. Points marked by red shapes correspond to the time series in B. (B) Time series demonstrating exemplar simulated cells for the regimes marked in A. The red shapes to the right of the time series correspond to the location in parameter space in A. (C) Heatmap of spikes per cluster in the underlying deterministic system. In the colourbar, ‘SS’ refers to a steady state, ‘T’ refers to tonic firing, ‘C’ refers to chaotic/irregular firing, and integers indicate number of spikes per cluster. (D) Time series demonstrating the deterministic dynamics underlying the stochastic traces in B. The red shapes to the right of the time series correspond to the location in parameter space in C. (For interpretation of the references to colour in this figure legend, the reader is referred to the web version of this article.)

To do so, we simulated 10 model neurons for 20 s over a range of values of $g_{\text{AHP}}$ and $g_{\text{h}}$. *P_C_*, which quantifies the proportion of clustered firing (see Section 2 and Fig. S1), was calculated for each parameter set. A summary of our results depicted as a heatmap of *P_C_* values and illustrated via exemplar membrane potential traces is shown in Fig. 1(A) and (B). For low values of $g_{\text{h}},$ the model cells only fire sporadic action potentials due to noise occasionally bringing the membrane potential above threshold (dark blue regions in Fig. 1(A)). For very low $g_{\text{AHP}},$ as $g_{\text{h}}$ is increased the system moves into a regime of tonic firing (yellow region in Fig. 1(A)). For intermediate values of $g_{\text{AHP}},$ as $g_{\text{h}}$ is increased clustered parameter regimes occur (orange regions in Fig. 1(A)) . For values of $g_{\text{AHP}}$ sufficiently high for clustering to occur, as $g_{\text{h}}$ is increased the system moves from very low *P_C_* towards a peak at *P_C_* ≈ 0.8, and then back down to lower *P_C_* (Fig. 1(A)). Therefore, spontaneous activity in the model arises due to a combination of noise and the applied current. Time courses associated with these values can be seen in Fig. 1(B). For these simulations, noise variance was set to σ/C=0.135 mV · ms${}^{-1}$ (see Section 2). Fig. S3 demonstrates that these results are robust to different values of noise, with noise values scaling *P_C_* in the clustered regimes. The effect of noise on *P_C_* for a single parameter regime is shown in Fig. S2.

In order to understand these dynamics, the deterministic system was also simulated over the same range of parameters. A heatmap representing the number of spikes per cluster and exemplar membrane potential traces are plotted in Fig. 1(C) and (D). To directly compare the dynamics of the deterministic system to the stochastic system, in Fig. S4 we present the heatmap of the deterministic system juxtaposed with heatmaps for the stochastic system at three different levels of noise variance. It can be seen in Fig. S4 that the heatmaps for the deterministic and stochastic system appear qualitatively similar in terms of the number of spikes per cluster (similar positioning of coloured regions in the heatmaps). In order to quantify this similarity we calculated the Pearson’s correlation between the number of spikes per cluster in the simulations of the deterministic system with the average number of spikes per cluster in the stochastic system. These values, which are indicated in the left hand corner of panels B–D of Fig. S4, were above 0.86, suggesting that an understanding of the deterministic clustering dynamics can be informative for understanding the clustering dynamics of the stochastic system.

**Fig. 2:**
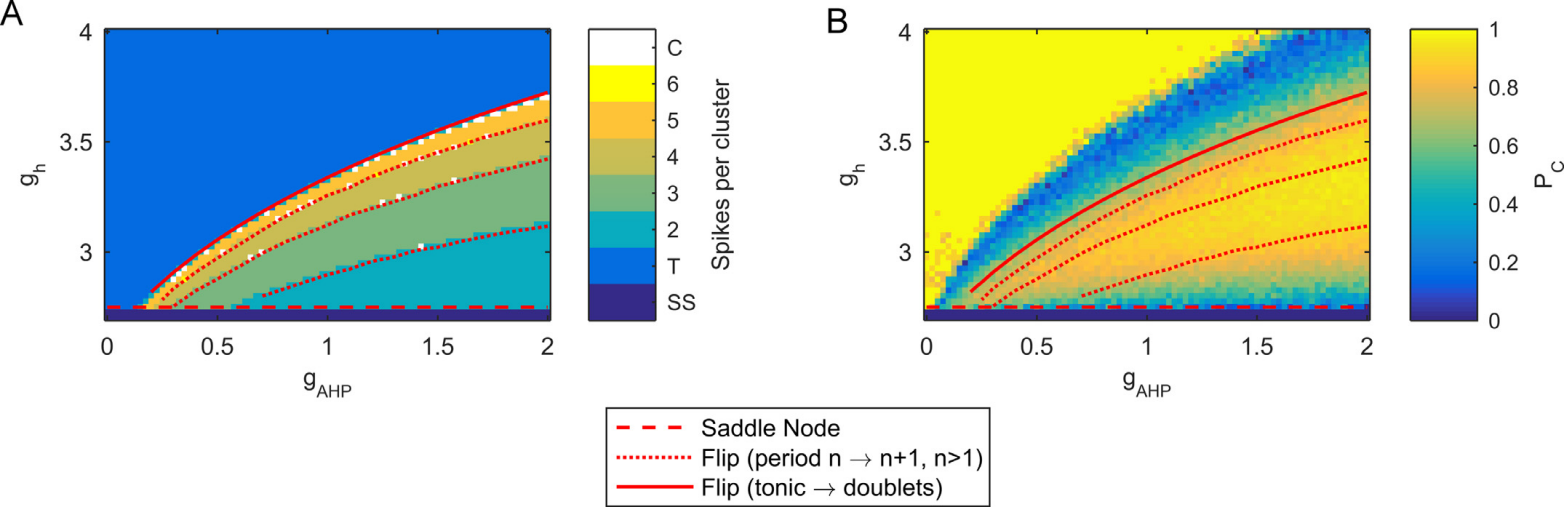
Bifurcations in two parameter space. (A) The heatmaps from Fig. 1(C) is overlayed with lines indicating locations of bifurcations in the deterministic system as $g_{\text{AHP}}$ and $g_{\text{h}}$ are varied. The dashed red line represents the location of a saddle node bifurcation. Dotted red lines show flip bifurcations that move the system from a period *n* to a period n+1 orbit, for *n* > 1. The solid red line shows a flip bifurcation that moves the system from tonic firing to period 2 firing, before transitioning into a period adding cascade. (B) The same bifurcations are overlayed on the *P_C_* heatmap of Fig. 1(A) to enable a visualisation of the behaviour of the stochastic system relative to the bifurcations in the deterministic system. (For interpretation of the references to colour in this figure legend, the reader is referred to the web version of this article.)

A two-parameter bifurcation analysis was performed over *g*_h_ and *g*_AHP_ (Fig. 2). For low values of *g*_h_, the deterministic system is in a stable steady state. This corresponds to the region of subthreshold sporadic excitability that generates occasional spiking in the stochastic system. As *g*_h_ is increased, a homoclinic bifurcation occurs at $g_{h}^{\text{HC}}=2.5477$, resulting in bistability between the steady state and a periodic orbit. This periodic orbit may be either period 1 (corresponding to tonic action potentials) or period  > 1 (corresponsing to clustering in the stochastic system) depending on *g*_AHP_. As *g*_h_ is increased further to $g_{\text{h}}^{\text{SN}}=2.7484$, the stable steady state collides with an unstable steady state in a saddle node bifurcation, resulting in periodic solutions corresponding to action potential firing being the only stable solutions. The location of the saddle node and homoclinic bifurcations are independent of *g*_AHP_. The saddle node bifurcation $g_{\text{h}}^{\text{SN}}$ is indicated by a dashed red line in two parameter space in Fig. 2.

**Fig. 3:**
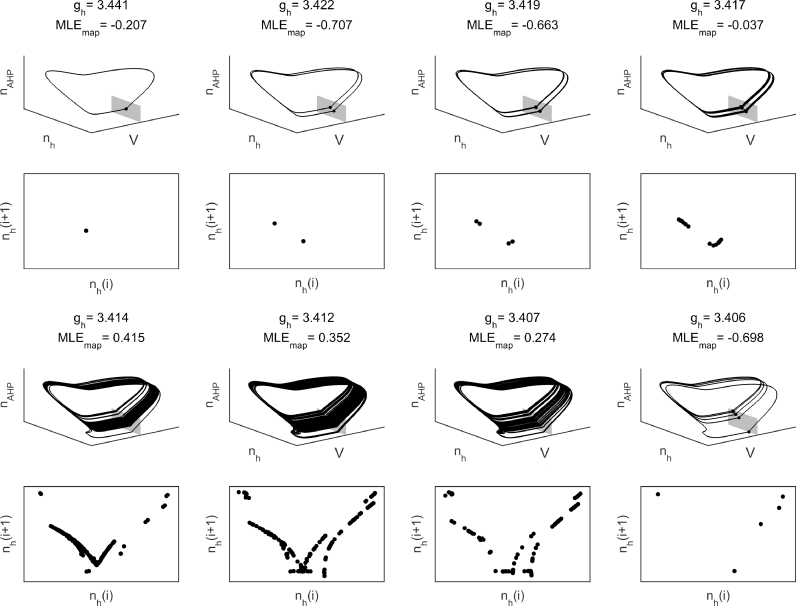
Transition from tonic firing to period 5 bursting. Each column represents a different parameter value as $g_{\text{h}}$ is decreased. For all simulations, $g_{\text{AHP}}=1.2$ and all other parameters are those in Table 1. Numbers shown at the top of each column are value of $g_{\text{h}}$ and maximum Lyapunov exponent on the map (${\text{MLE}}_{\text{map}}$). ${\text{MLE}}_{\text{map}}>0$ represents chaos. For each parameter value, the top row demonstrates the flow in the (*V, n*_h_, *n*_AHP_) subspace about the Poincaré section V=0 (shaded in grey) and the bottom row is the Poincaré return map for *n*_h_. For the chaotic regimes, the system was simulated for 30 s to reach the attractor and then a further 30 s of simulations are shown. From a tonic regime, as $g_{\text{h}}$ is decreased the system undergoes a flip cascade into chaos before transitioning into a period 5 (bursting) orbit.

For $g_{\text{h}}>g_{\text{h}}^{\text{SN}},$ only a stable periodic orbit exists, generated by the homoclinic bifurcation at $g_{\text{h}}^{\text{HC}}$. Orbits with a range of number of spikes per period can be found beyond this bifurcation. Period 1 orbits correspond to tonic action potentials, whilst period  > 1 orbits correspond to firing in multiplets, i.e. bursting. By comparing Fig. 2(A) and (B), one can observe that the regimes of period  > 1 in the deterministic system correspond to clustered action potential firing in the stochastic system. The transitions between orbits of different periods (e.g., from period 2 doublets to period 3 triplets) occur via flip bifurcations (Barrio, Shilnikov, 2011, Channell, Cymbalyuk, Shilnikov, 2007), drawn in Fig. 2 by dotted red lines. The transition between period 1 orbits (tonic spiking) and orbits with period  > 1 (bursting) is indicated by a solid red line in Fig. 2. Seen in terms of decreasing values of $g_{\text{h}},$ the bifurcation underlying this transition is a flip bifurcation of the period 1 orbit into period 2 regime. As $g_{\text{h}}$ is decreased further, the system undergoes a flip or spike adding cascade into chaotic dynamics, before a stable period 5 orbit is established. Poincaré return maps and Lyapunov exponents demonstrating an example of this transition are shown in Fig. 3.

**Table 1:** Parameters used in the model.

Parameter	Value	Parameter	Value
*C*	1.46 *μ* F · cm${}^{-2}$	$g_{\text{NaT}}$	24 mS · cm${}^{-2}$
*I*_app_	0.3 *μ* A · cm${}^{-2}$	$g_{\text{NaP}}$	0.075 mS · cm${}^{-2}$
$E_{\text{Na}}$	55 mV	$g_{\text{Kdr}}$	11 mS · cm${}^{-2}$
$E_{\text{K}}$	−85 mV	$g_{\text{Kaf}}$	0.1 mS · cm${}^{-2}$
$E_{\text{h}}$	−30 mV	$g_{\text{Kas}}$	0.5 mS · cm${}^{-2}$
$E_{\text{L}}$	−88.5 mV	$g_{\text{L}}$	0.15 mS · cm${}^{-2}$

Moving beyond this bifurcation to high values of $g_{\text{h}}$ and low values of $g_{\text{AHP}}$ yields *P_C_* ≈ 1 in the stochastic system. This observation could be explained by a highly stable periodic orbit and therefore diminished effects of noise. However, in this case a high value of clustering arises due to the way *P_C_* is calculated, essentially tonic firing with an ISI  < 250 ms is classified as a single cluster (Fig. S1). As the flip bifurcation is approached from above and left, the orbit becomes less stable allowing noisy perturbations to cause deviations away from individual action potentials. This induces quiescent intervals that become large enough to fall in the range [250,300] ms, thus causing the *P_C_* value to drop substantially in magnitude, giving rise to the light blue upper region of low *P_C_* in Fig. 1(A).

**Fig. 4:**
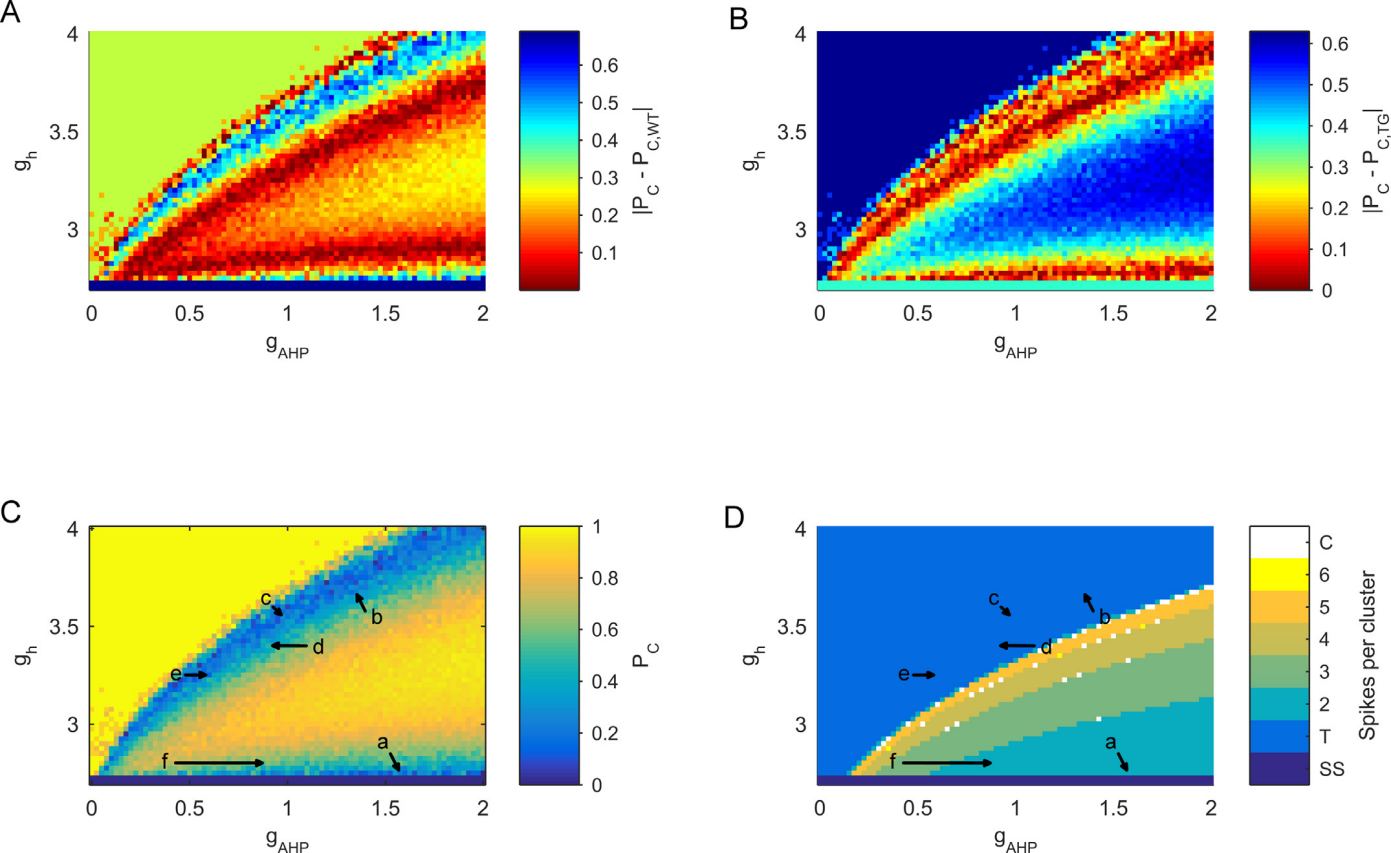
Paths through parameter space that can result in reduced clustering observed in the rTg4510 model of dementia (A) Heatmap of $|P_{C}-P_{C,WT}|,$ where $P_{C,WT}=0.69$ is the mean value of clustering seen in dorsal mEC-SCs in wild type animals (Booth et al., 2016). Red indicates regions in which *P_C_* of the model is close to *P*_*C, WT*_, whereas blue indicates regions where the model is farthest from *P*_*C, WT*_. (B) Heatmap of $|P_{C}-P_{C,TG}|,$ where $P_{C,TG}=0.37$ is the mean value of clustering seen in dorsal mEC-SCs in rTg4510 transgenic (i.e. dementia) animals (Booth et al., 2016). (C) The heatmap of Fig. 1(A) is overlayed with arrows indicating potential paths through the $\left(g_{\text{AHP}},g_{\text{h}}\right)$ parameter space that could lead to the changes in *P_C_* observed in the rTg4510 experimental model. (D) The heatmap of Fig. 1(C) is overlayed with arrows indicating potential paths through the $\left(g_{\text{AHP}},g_{\text{h}}\right)$ parameter space that could lead to the changes in *P_C_* observed in the rTg4510 experimental model. (For interpretation of the references to colour in this figure legend, the reader is referred to the web version of this article.)

Experimental observations have shown dorsal *P_C_* to be approximately 0.69 in healthy animals and approximately 0.37 in rTg4510 transgenic animals (Booth et al., 2016). We used these values to define possible paths through parameter space that may account for differences observed in rTg4510 (Fig. 4). Given that experimental recordings found no differences in $I_{\text{h}}$ but found differences in AHP amplitude (Booth et al., 2016), paths E and F in Fig. 4(C) and (D) are the most likely changes in parameter space occurring in rTg4510. The dynamics of path F recreate firing patterns seen in data most realistically, since firing frequency in parameter sets in path E is much higher than in data (Booth et al., 2016). This could be explained by the fact that in path E, clustering arises due to noise cancelling action potentials in a tonic firing regime, as opposed to underlying dynamics causing clustered firing. Path F suggests that the underlying noise-free system is undergoing a flip bifurcation from period 3 bursts to period 2 bursts, resulting in the reduced clustering seen in rTg4510.

### 3.2 Fast-slow analysis of deterministic clustering

**Fig. 5:**
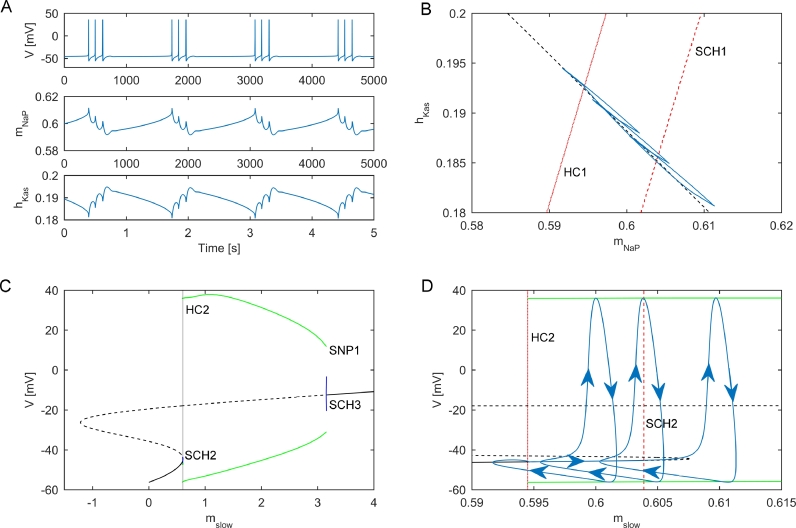
Fast-slow analysis of deterministic bursting (A) Membrane potential (top) and slow variables ($m_{\text{NaP}},$ middle and $h_{\text{Kas}},$ bottom) through four cycles of bursting in the deterministic system. (B) Bifurcations in the fast subsystem overlayed on the model trajectory in the $\left(m_{\text{NaP}},h_{\text{Kas}}\right)$ plane. The red dashed line indicates a subcritical Hopf bifurcation (SCH1), whereas the dotted red line indicates a homoclinic bifurcation (HC1). The black dashed line shows the linear model that combines $h_{\text{Kas}}$ and $m_{\text{NaP}}$ into a single slow variable, $m_{\text{slow}}$. (C) Bifurcation analysis of the fast subsystem of the model using $m_{\text{slow}}$ as a bifurcation parameter. A stable equilibrium (solid black line) is shown to lose stability (dashed black line) via a subcritical Hopf bifurcation (SCH2). The stable periodic orbit (solid green line) disappears in a homoclinic bifurcation (HC2). A region of bistability exists (shaded region, zoomed in panel D). See text for a description of the remaining bifurcations. (D) A close up of the bifurcations occurring in the region of bistability shown in grey in panel C. The blue line indicates a trajectory of the full system through a single period of bursting, with arrows indicating the direction of time. Dashed and dotted red lines correspond to the bifurcations of the fast subsystem introduced in panel B. (For interpretation of the references to colour in this figure legend, the reader is referred to the web version of this article.)

The analysis above suggests that clustered firing patterns may arise due to noise perturbations to a periodic bursting regime. In order to further understand these dynamics, a fast-slow analysis was performed on the deterministic system within this regime. We chose parameters to be $g_{\text{AHP}}=0.425$ and $g_{\text{h}}=2.8,$ which results in periodic bursts of three action potentials. We first examined simulations, which revealed two variables operating with a slow time scale, namely $m_{\text{NaP}}$ and $h_{\text{Kas}}$ (Fig. 5(A)). Keeping the two slow variables fixed, the remaining (fast) subsystem was subjected to a numerical bifurcation analysis, which revealed two bifurcations of importance for describing the bursting dynamics (see Fig. 5(B)). For low values of $m_{\text{NaP}},$ there exists a stable steady state which loses stability via a subcritical Hopf bifurcation (denoted SCH1) as $m_{\text{NaP}}$ is increased (marked by a dashed red line in Fig. 5(B)). For high values of $m_{\text{NaP}}$ there exists a stable periodic orbit of period 1, which disappears via a homoclinic bifurcation (denoted HC1 and marked by a dotted red line in Fig. 5(B)) as $m_{\text{NaP}}$ is decreased. Between these two bifurcations there is a region of bistability between the steady state and the periodic orbit. These bifurcations in $m_{\text{NaP}}$ are drawn over a range of values of $h_{\text{Kas}}$ in Fig. 5(B). A full bifurcation diagram and example bistable region for $m_{\text{NaP}}$ for $h_{\text{Kas}}=0.19$ is shown in Fig. S5.

Plotting the periodic solution of the full subsystem in the two variables ($m_{\text{NaP}}$ and $h_{\text{Kas}},$ Fig. 5(B)) is sufficient to describe the bursting dynamics. The trajectory follows a hysteresis loop through the fast subsystem. Beginning in the quiescent period between bursts, the two slow variables will be at a position in phase space such that the fast subsystem is on the steady state branch. The periodic solution’s trajectory then moves along the steady state branch until SCH1 is reached, at which point the fast subsystem moves to the periodic orbit branch. This initiates the burst, with action potentials firing while slow variables move along the periodic orbit branch towards HC1. Once HC1 is reached, the burst ends as the fast subsystem returns to the steady state branch.

Fig. 5(B) suggests that the slow system can be reduced to a single slow variable $m_{\text{slow}}$ with the approximation $m_{\text{NaP}}=m_{\text{slow}}$ and $h_{\text{Kas}}=-0.7657m_{\text{slow}}+0.6477$. This linear approximation of the two slow variables is shown in Fig. 5(B). The full bifurcation diagram for the fast subsystem as $m_{\text{slow}}$ is varied is shown in Fig. 5(C). As before, the stable steady state is lost via subcritical Hopf bifurcation (SCH2), and the stable periodic orbit is lost via homoclinic bifurcation (HC2). Fig. 5(C) shows the remaining bifurcations. The unstable periodic orbit generated by SCH2 is lost via a homoclinic (HC3). The unstable steady state following SCH2 becomes stable via another subcritical Hopf (SCH3). The unstable periodic orbit generated by SCH3 collides with the stable periodic orbit generated in HC2 and both periodic orbits disappear via a saddle node of periodics (SNP1). As in the case of the two dimensional slow subsystem, there is bistability between the stable equilibrium and the stable periodic orbit (Fig. 5(D)) resulting in traditional fast-slow hysteresis loop bursting. The trajectory of a single burst is shown in Fig. 5(D).

### 3.3 Subthreshold dynamics

In order to validate the model, we tested whether it reproduced experimental results that were not used in the development of the model; i.e. when choosing parameter regimes that allow for mEC-SC-like clustering dynamics. Subthreshold oscillations in the theta (4–12 Hz) range are another key electrophysiological feature of mEC-SCs, so in this section we explore whether theta band subthreshold activity appears in the model.

**Fig. 6:**
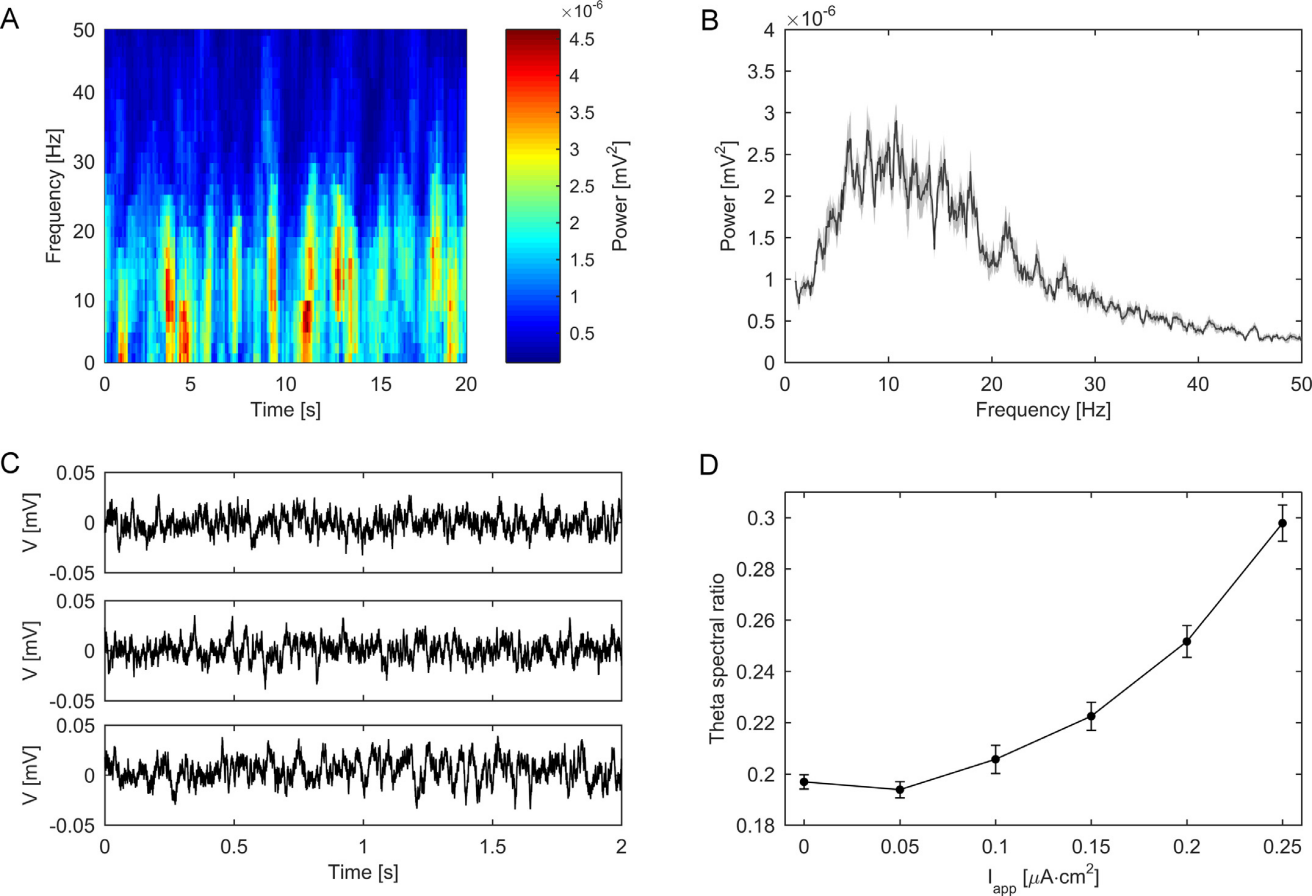
Analysis of subthreshold oscillations (A) Spectrogram of exemplar 20 s subthreshold simulations. (B) Power spectrum of 20s simulations (averaged over 10 cells). The shaded region shows standard error. (C–E) Exemplar simulations with $I_{\text{app}}=0.05\;$*μ* A (C), 0.15 *μ* A (D), and 0.25 $\mu A\cdot {\text{cm}}^{-2}$ (E). (F) Theta spectral ratio, defined as the ratio of total theta power to total broadband (1–300 Hz) power, plotted as a function of *I*_app_.

The bottom trace of Fig. 1(B) demonstrates the noise driven response of the model in its subthreshold regime. mEC-SCs have been shown to generate subthreshold membrane potential fluctuations with dominant frequencies in the theta band (Alonso and Klink, 1993). We therefore quantified the power spectrum of dynamics generated by our noise driven system. The stochastic system, with parameters chosen as in Section 3.2, *I*_app_ set below action potential threshold (0.25 *μ* A · cm${}^{-2}$), and white noise added to the membrane potential, was simulated for 20 s with low noise variance (σ/C=0.005 mV · ms${}^{-1}$). Fig. 5(D) shows an example spectrogram, demonstrating high power between 0–20 Hz with a peak in the theta (4–12 Hz) range. The mean power spectrum over an ensemble of simulations (Fig. 6(B)) shows peak power to be in the theta band, with peak frequency found to be at 10.40 ± 1.09 Hz (mean  ±  standard error). Whilst low noise variance was used in these simulations in order to elucidate mechanisms, Fig. S6 shows simulations using the same amount of noise as in previous sections (σ/C=0.135 mV · ms${}^{-1}$) to demonstrate that theta range fluctuations still arise in system with more realistic noise levels.

To further understand the origin of this subthreshold preferential theta power, we analysed the deterministic system. Fig. S7 shows a bifurcation diagram in *I*_app_. The deterministic system undergoes a saddle node bifurcation at $I_{\text{app}}^{\text{SN}}=0.2738\;\mu$A · cm${}^{-2}$; for $I_{\text{app}}<I_{\text{app}}^{\text{SN}}$ a stable steady state exists. A supercritical Hopf bifurcation occurs at $I_{\text{app}}^{\text{Hopf}}=42.10\;\mu$A · cm${}^{-2}$, generating a stable periodic orbit that is lost via a homoclinic bifurcation at $I_{\text{app}}^{\text{HC}}=0.2401\;\mu$A · cm${}^{-2}$ demonstrating bistability between spiking and steady state in the range $I_{\text{app}}^{\text{HC}}<I_{\text{app}}<I_{\text{app}}^{\text{SN}}$. No other Hopf bifurcations occur in *I*_app_, hence the deterministic system does not exhibit stable subthreshold oscillations within this parameter regime. We note that noise perturbations can drive the membrane potential above threshold even for $I_{\text{app}}<I_{\text{app}}^{\text{SN}}$ (see Fig. S8 for analysis of spike onset in relation to injected current and differing noise variance). This justifies our choice of $I_{\text{app}}=0.25\;\mu$A · cm${}^{-2}$ as this is sufficiently below threshold that no action potentials are observed.

In the absence of noise, the system is in a steady state and therefore no deterministic theta band oscillations arise. A potential mechanism by which white noise on a steady state can result in power spectral peaks is if the steady state is a focus. The resonant frequency of a focus can be calculated as the imaginary part of the complex conjugate eigenvalues of the Jacobian normalised by a value of 2*π*. A pair of complex conjugate eigenvalues demonstrated that the steady state is a focus with a resonant frequency of 6.32 Hz. The effect of changing applied current was also tested (Fig. 6(C) and (D)). In experimental recordings, theta power is seen to increase as *I*_app_ approaches threshold for action potential generation (Alonso and Klink, 1993). Fig. 6(C) shows time series traces for a range of values of *I*_app_, demonstrating theta power increasing as *I*_app_ is increased. Theta band spectral ratio was calculated as the ratio of total power in the theta band to total power in the 1–300 Hz broad band, shown in Fig. 6(D). Total power in the delta (1–3 Hz), theta (4–12 Hz), beta (15–30 Hz) and gamma (30–300 Hz), normalised by width of band, is shown in Fig S9. Each of these figures demonstrate the clear emergence of peak theta power as *I*_app_ is increased and threshold is approached. A Kruskal–Wallis test confirms a significant effect of applied current on spectral ratio ($\chi^{2}=44.97,$ $p=1.47\times 10^{-8}$).

## 4 Discussion

In this study we analysed a conductance based model of a layer II medial entorhinal cortex stellate cell (mEC-SC), demonstrating that it is capable of generating clustered action potential firing with a range of quantitative *P_C_* values that are observed in experiments. We demonstrated that these dynamics arise due to a subcritical Hopf/homoclinic bursting mechanism, which causes multiple period limit cycles that when perturbed by extrinsic noise display action potential clustering. We further demonstrated that the same model can generate experimentally observed subthreshold membrane potential fluctuations with power spectral peak in the theta band.

### 4.1 Derivation of the model, approximation of noise, and relationship to the Markov chain model

Dudman and Nolan (2009) presented a biophysically realistic Markov chain (MC) gated model of enthorhinal cortex stellate cells. MC models account for random fluctuations in the opening and closing of ion channels intrinsic to neurons (Goldwyn, Imennov, Famulare, Shea-Brown, 2011, White, Rubinstein, Kay, 2000) by assigning them a voltage dependent probability of opening or closing. However, dynamic analysis of Markov chain models is challenging. Furthermore, Markov chain models are computationally expensive. For these reasons, in this paper, the MC gated model was converted to the deterministic Hodgkin-Huxley formulation for ion channel gates (Eq (1); Hodgkin and Huxley, 1952) under the assumption that the number of ion channels is sufficiently high that a density approximation can be justified, resulting in a system of ordinary differential equations (ODEs). Channel noise in the neuron was not explicitly modelled, but approximated by extrinsic, Gaussian noise on the membrane potential. We demonstrated that this was sufficient to produce clustered action potential dynamics and theta range subthreshold fluctuations in line with experiments (Alonso and Klink, 1993).

### 4.2 Action potential clustering

Clustered action potential firing, in which two or more action potentials are fired in succession before a long quiescent period, is a feature of *in vitro* recordings of layer II medial entorhinal cortex stellate cells. Action potential clustering is hypothesised to depend on the AHP and $I_{\text{h}}$ currents based on computational studies and correlated gradients in dynamics associated with these currents (Booth, Ridler, Murray, Ward, de Groot, Goodfellow, Phillips, Randall, Brown, 2016, Fransén, Alonso, Dickson, Magistretti, Hasselmo, 2004, Garden, Dodson, O’Donnell, White, Nolan, 2008, Giocomo, Hasselmo, 2008, Giocomo, Hasselmo, 2009, Nolan, Dudman, Dodson, Santoro, 2007, Pastoll, Ramsden, Nolan, 2012, Yoshida, Jochems, Hasselmo, 2013). Motivated by this, the dependence of these two parameters on clustering was tested in the model. A two parameter bifurcation analysis (Fig.1(A)) demonstrated that regions of quiescence, tonic firing, and clustered firing coexist. Furthermore, a range of values of *P_C_* were found, allowing for control over the amount of clustering in the model.

Analysis of the deterministic model allowed for understanding of the mechanisms behind clustering (Fig. 1(C)). Regions corresponding to tonic firing in the stochastic model correspond to regions of tonic firing in the deterministic model. As the regions of clustering are approached from the regions of tonic firing, a period doubling cascade occurs until stable multiplets (‘bursts’ of action potentials) are reached. Flip bifurcations (Barrio, Shilnikov, 2011, Channell, Cymbalyuk, Shilnikov, 2007) occur, changing the number of spikes per burst. Eventually, firing is lost althogether via a homoclinic bifurcation as $g_{\text{h}}$ is decreased. It is worth noting that a region of bistability exists before the homoclinic is reached in which the stable periodic orbit coexists with a stable steady state. In this region of bistability, it was found that simulations of the stochastic system starting on or near the periodic orbit are soon driven by noise towards the stable steady state, and hence sustained action potential firing in this region of the stochastic system is rare. Similar results occur for changes in *I*_app_ if $g_{\text{h}}$ is held constant in certain parameter regimes (Fig. S7), reflecting results in data that increasing applied current will increase number of spikes per cluster before moving the system into tonic firing (Alonso and Klink, 1993). This suggests that the different dynamics due to alterations in $g_{\text{h}}$ may arise because of a change in resting membrane potential as $g_{\text{h}}$ is varied. No such change in resting membrane potential is observed as $g_{\text{AHP}}$ is altered. Analysis of a bursting regime demonstrated that bursting arises due to a fast-slow mechanism in which two slow variables drive the fast subsystem through a hysteresis loop. In terms of bifurcations in the fast sub-system, the bursting mechanism in this model can be classified as subcritical Hopf/homoclinic type (Izhikevich, 2000).

The generation of clustered action potential firing by deterministic, periodic bursting perturbed by extrinsic noise differs from past interpretations of clustering. In the Markov chain formalism of the model, Dudman and Nolan (2009) suggested clustering was the result of a transient increase in probability of firing during recovery from the AHP due to the stochastic mechanisms, and they demonstrated that clustering was not possible in the deterministic version of the model. In our study, we systematically explored the consequences of changing $g_{\text{h}}$ and $g_{\text{AHP}},$ and found different dynamic regimes in the deterministic system, including steady state and tonic firing regimes that do not correspond to clustered firing in the stochastic model. It is possible that further exploration of the dynamics of the model of Dudman and Nolan (2009) would reveal similar bursting regimes to those reported herein. Although experimental verification of these interpretations is difficult, there are some agreements in mechanisms between these two models, however. The effect of changing $g_{\text{AHP}}$ in the MC model has not been studied, but within a clustered parameter regime the affect of reducing $g_{\text{h}}$ in the SDE model largely agrees with the results of reducing $g_{\text{h}}$ in the MC model - a reduced value of *P_C_*. The interpretation of increased probability of firing during recovery from AHP also emphasises the importance of the AHP current in clustering in the MC model.

A number of other parameters are likely to play a role in clustering. AHP halfwidth and $I_{\text{h}}$ time constants may be important, as dorsoventral gradients in these properties also correspond to gradients in clustering (Boehlen, Heinemann, Erchova, 2010, Booth, Ridler, Murray, Ward, de Groot, Goodfellow, Phillips, Randall, Brown, 2016, Giocomo, Hasselmo, 2008, Giocomo, Hasselmo, 2009, Giocomo, Zilli, Fransen, Hasselmo, 2007, Pastoll, Ramsden, Nolan, 2012), but these have not been studied here. Figs. S2 and S3 demonstrate that the variance of noise chosen will also dictate the amount of clustering; increasing noise variance increases the likelihood of sporadic spiking or action potential cancellation, thus affecting the patterned firing.

### 4.3 Subthreshold theta resonance

Stellate cells in Layer II of the medial entorhinal cortex are known to exhibit subthreshold oscillations in the theta (4–12 Hz) range that increase in power as action potential threshold is approached (Alonso and Klink, 1993). It is believed these subthreshold oscillations are noise driven (White et al., 1998). In our deterministic (noise free) model, subthreshold oscillations do not exist, since we operated in a steady-state regime. However, the steady state is a focus with resonant frequency of 6.32 Hz, suggesting that with the addition of noise, a spectrum with preferential power in the theta band may arise. We found that a small amount of white noise on the membrane potential is sufficient to give rise to subthreshold dynamics with multiple peaks within the theta range and peak power at around 10 Hz. The difference in peak frequency found in simulations compared to the prediction from the linearisation of the focus may be due to noise in the simulated spectrum as well as noise induced frequency shifts (Bonnin and Corinto, 2013). Furthermore it was shown that the relative power in the theta band is significantly larger close to threshold than far below threshold.

To model the dynamics of subthreshold activity of stellate cells, two classes of model have previously been proposed. The first class of model utilises noisy perturbations to deterministic limit cycle dynamics. In this case, the output of the deterministic model would be regular, periodic oscillations and the related stochastic model would exhibit strongly periodic dynamics contaminated by noise. Previous models of subthreshold oscillations in stellate cells that fall into this class include (Dickson, Magistretti, Shalinsky, Fransén, Hasselmo, Alonso, 2000, Fransén, Alonso, Dickson, Magistretti, Hasselmo, 2004, Rotstein, Oppermann, White, Kopell, 2006, White, Budde, Kay, 1995). In the second class of model, such as the one presented in this study and the Izhikevich (2007), theta band fluctuations arise due to noisy perturbations on a focus steady state, which results in a resonant response. In contrast to the aforementioned class of limit cycle models, fluctuations exist only in the presence of noise. Furthermore, in the noisy focus class of model, the dynamics appear less obviously periodic than in limit cycle models, resembling a stochastic process with peak power in the theta range. Experimental and modelling studies have suggested that removing channel noise results in loss of subthreshold oscillations (Dorval, White, 2005, Dudman, Nolan, 2009, White, Klink, Alonso, Kay, 1998) and that stellate cell subthreshold dynamics are more reflective of a stochastic process with theta peak than a periodic process with additive noise (Dodson et al., 2011). These results are consistent with the noisy focus class of model, which the model we present belongs to. However, we note that the mechanisms of the two classes of model are closely related, since in theory, one expects to find a focus steady state close to a Hopf bifurcation into a limit cycle (White et al., 1995) with resonant frequency close to that of the limit cycle.

For biological insight into the currents involved in the generation of subthreshold limit cycles or resonance, reduced models, which remove currents that are predominantly active during action potential initiation or recovery, can be of interest. $I_{\text{h}}+I_{\text{NaP}}+I_{\text{L}}$ models have been shown to generate theta band limit cycle oscillations (Dickson, Magistretti, Shalinsky, Fransén, Hasselmo, Alonso, 2000, Fransén, Alonso, Dickson, Magistretti, Hasselmo, 2004, Rotstein, Oppermann, White, Kopell, 2006). As discussed above, the alternative mechanisms of noise-perturbed focus and limit cycle dynamics are related, so it is of interest to test whether making similar reductions in our model maintains the theta band resonance. Setting all currents but $I_{\text{h}}$ and $I_{\text{NaP}}$ to their steady state value, we found that the corresponding steady state becomes a node and hence theta band resonance is lost. A detailed study of the mechanisms underlying the noise response of our model is an avenue for future work.

### 4.4 Implications for dementia

The entorhinal cortex is one of the first areas to be affected in dementias featuring a tau pathology such as Alzheimer’s disease (Braak and Braak, 1991). In the rTg4510 mouse model of tauopathy, dorsoventral gradients in action potential clustering in layer II entorhinal cortex stellate cells were abolished (Booth et al., 2016). A motivating application for a mathematical model of mEC-SCs in which action potential clustering can be controlled is to understand the mechanisms behind the dysfunction in clustered firing in animal models of dementia. Future work will involve exploring this relationship in more detail, but some key points can be stated from the work presented here. In the wild type animals, dorsal mEC-SCs fired highly clustered action potentials. This clustering was greatly reduced in the rTg4510 animals. Whilst $I_{\text{h}}$ mediated sag amplitude was unaffected (suggesting no changes in $g_{\text{h}}$), an increase in amplitude of the AHP was seen in rTg4510 dorsal cells. The AHP amplitude, which scales with AHP conductance, has been demonstrated to be mechanistically related to *P_C_* in this model and previous studies (Fernandez, White, 2008, Fransén, Alonso, Dickson, Magistretti, Hasselmo, 2004). A possible mechanism for the reduced *P_C_* in rTg4510 is an increase in $g_{\text{AHP}},$ resulting in the system undergoing a flip bifurcation resulting in fewer spikes per cluster. An example of this is the path through parameter space marked F in Fig. 4, which results in realistic mEC-SC like clustering dynamics, with a change in parameters that reflects those seen in rTg4510. Future work will involve fitting parameters to the data to explore this in more detail.

Network activity was also seen to be disrupted in rTg4510 (Booth et al., 2016). Dorsoventral gradients in phase-amplitude coupling (PAC) between theta and gamma rhythms in the local field potential was found to be disrupted in rTg4510 animals. Similar to clustering patterns, dorsoventral gradients in PAC were disrupted. Networks of modelled stellate cells, spatially extended along the dorsoventral axis, may be used to explore whether disruption in patterned action potential activity alone is sufficient to replicate deficiencies in PAC, or whether network properties such as dorsoventral gradients in inhibitory projections also come into play (Beed et al., 2013). Past computational studies of theta-gamma PAC have involved use of simple models that do not intrinsically fire in clusters such as the exponential integrate-and-fire (Solanka et al., 2015) or Hodgkin–Huxley (Wulff et al., 2009) models. Dorsoventral gradients in clustering intrinsic to cells cannot be studied using these models, and hence are not suitable to test whether intrinsic clustering is related to theta-gamma coupling. The model presented here is more suited to this type of study, as clustering can be controlled via biophysically realistic mechanisms.

### 4.5 Conclusions

In this work, we have presented a stochastic differential equation (SDE) model of Layer II medial entorhinal cortex stellate cells based on the Markov Chain formalism of the model presented by Dudman and Nolan (2009), but driven by extrinsic white noise to the membrane potential. We demonstrated that this model captures the key dynamics of mEC-SCs seen in electrophysiological recordings including subthreshold oscillations in the theta range and clustered action potential firing (Alonso and Klink, 1993). To understand the mechanisms underpinning clustered action potential firing, a numerical bifurcation analysis was performed on the underlying system of ordinary differential equations. Clustering was shown to arise due to flip bifurcations in the AHP and h-current conductance parameters, and is driven by two slow variables ($m_{\text{NaP}}$ and $h_{\text{Kas}}$) driving the remaining fast subsystem through a subHopf/homoclinic type hysteresis loop. Furthermore, exploration of parameter space demonstrates that control of the AHP and h-current conductances allows for control of *P_C_*, which quantifies the amount of action potential clustering exhibited by the model. The model provides an important tool for further understanding alterations to mEC spatiotemporal dynamics that arise in dementias featuring a tau pathology (Booth et al., 2016).

## Appendix A Model equations

The model contains a total of eight ionic currents. These are transient and persistent sodium currents ($I_{\text{NaT}}$ and $I_{\text{NaP}}$ respectively), a potassium delay rectifier ($I_{\text{Kdr}}$), fast and slow potassium A-type currents ($I_{\text{Kaf}}$ and $I_{\text{Kas}}$ respectively), a hyperpolarisation activated $I_{\text{h}}$ current, an ohmic leak ($I_{\text{L}}$) and finally a phenomenological after hyperpolarisation current that is dependent on the time since last spike ($I_{\text{AHP}}$).

The current balance equation for our model is given in Eq. (2), with each current represented as in Eq. (3). The fraction of open gates for each channel is given by

(A.1) $$\begin{array}{cc} \psi_{\text{NaT}} & =m_{\text{NaT}}^{3}h_{\text{NaT}} \end{array}$$

(A.2) $$\begin{array}{cc} \psi_{\text{NaP}} & =m_{\text{NaP}}h_{\text{NaP}} \end{array}$$

(A.3) $$\begin{array}{cc} \psi_{\text{Kdr}} & =n_{\text{Kdr}}^{4} \end{array}$$

(A.4) $$\begin{array}{cc} \psi_{\text{Kaf}} & =m_{\text{Kaf}}h_{\text{Kaf}} \end{array}$$

(A.5) $$\begin{array}{cc} \psi_{\text{Kas}} & =m_{\text{Kas}}h_{\text{Kas}} \end{array}$$

(A.6) $$\begin{array}{cc} \psi_{\text{h}} & =n_{\text{h}} \end{array}$$

(A.7) $$\begin{array}{cc} \psi_{\text{AHP}} & =n_{\text{AHP}}^{3} \end{array}$$

(A.8) $$\begin{array}{cc} \psi_{\text{L}} & =1 \end{array}$$

where gating variables are given by the system of ODEs in Eq. (1). Probabilities of gates opening and closing are given by the functions

(A.9) $$\begin{array}{cccc} \alpha_{m_{\text{NaT}}} & =\frac{0.38(V+33)}{1-e^{\frac{V+33}{9}}}, & \beta_{m_{\text{NaT}}} & =\frac{-2.3(V+58)}{1-e^{\frac{V+58}{12}}} \end{array}$$

(A.10) $$\begin{array}{cccc} \alpha_{h_{\text{NaT}}} & =\frac{-0.03(V+48)}{1-e^{\frac{V+48}{12}}}, & \beta_{h_{\text{NaT}}} & =\frac{0.05(V+21)}{1-e^{\frac{V+21}{9}}} \end{array}$$

(A.11) $$\begin{array}{ccc} \alpha_{m_{\text{NaP}}} & = & \frac{1.6*10^{-4}(0.38\left(V+64.409\right)}{1-e^{-0.38023(V+64.409)}},\\ \beta_{h_{\text{NaP}}} & = & \frac{1.2*10^{-4}(-0.216\left(V+17.014\right)}{1-e^{0.21598(V+17.014)}} \end{array}$$

(A.12) $$\begin{array}{cccc} \alpha_{h_{\text{NaP}}} & =\frac{1.5}{1+e^{\frac{-42.1-V}{3}}}, & \beta_{m_{\text{NaP}}} & =\frac{1}{1+e^{\frac{42.1-V}{3}}} \end{array}$$

(A.13) $$\begin{array}{cccc} \alpha_{n_{\text{Kdr}}} & =\frac{0.02(V+38)}{1-e^{\frac{V+38}{10}}}, & \beta_{n_{\text{Kdr}}} & =\frac{-0.018(V+47)}{1-e^{\frac{V+47}{35}}} \end{array}$$

(A.14) $$\begin{array}{cccc} \alpha_{m_{\text{Kaf}}} & =\frac{0.01(V+18.3)}{1-e^{-0.067(V+18.3)}}, & \beta_{m_{\text{Kaf}}} & =\frac{-0.01(V+18.3)}{1-e^{0.067(V+18.3)}} \end{array}$$

(A.15) $$\begin{array}{cccc} \alpha_{h_{\text{Kaf}}} & =\frac{-0.01(V+58)}{1-e^{0.122(V+58)}}, & \beta_{h_{\text{Kaf}}} & =\frac{0.01(V+58)}{1-e^{-0.122(V+58)}} \end{array}$$

(A.16) $$\begin{array}{cccc} \alpha_{m_{\text{Kas}}} & =\frac{0.001(V+18.3)}{1-e^{-0.067(V+18.3)}}, & \beta_{m_{\text{Kas}}} & =\frac{-0.001(V+18.3)}{1-e^{0.067(V+18.3)}} \end{array}$$

(A.17) $$\begin{array}{cccc} \alpha_{h_{\text{Kas}}} & =\frac{-6.7*10^{-5}\left(V+58\right)}{1-e^{0.122(V+58)}}, & \beta_{h_{\text{Kas}}} & =\frac{-6.7*10^{-5}\left(V+58\right)}{1-e^{-0.122(V+58)}} \end{array}$$

(A.18) $$\begin{array}{cccc} \alpha_{n_{\text{h}}} & =\frac{18.3*10^{-3}}{1+e^{\frac{V+114.2}{20.33}}}, & \beta_{n_{\text{h}}} & =\frac{3.3*10^{-2}}{1+e^{\frac{V+51.5}{10.94}}} \end{array}$$

(A.19) $$\begin{array}{cccc} \alpha_{n_{\text{AHP}}} & =1.5e^{\frac{-(t-t_{\text{spike}})}{25}}, & \beta_{n_{\text{AHP}}} & =1.6 \end{array}$$

All equations are adapted from Dudman and Nolan (2009).

## Appendix B Supplementary materials

Supplementary Data S1 Supplementary Raw Research Data. This is open data under the CC BY license http://creativecommons.org/licenses/by/4.0/ Supplementary Data S1
