# Supplementary material for: Control of clustered action potential firing in a mathematical model of entorhinal cortex stellate cells
Source: J Theor Biol. 2018 Jul 14;449:23–34. doi: 10.1016/j.jtbi.2018.04.013 (PMC5947116; doi:10.1016/j.jtbi.2018.04.013)
Supplement: Supplementary Data S1 — Supplementary Raw Research Data. This is open data under the CC BY license http://creativecommons.org/licenses/by/4.0/ [file mmc1.pdf]

Supplementary Figure Captions

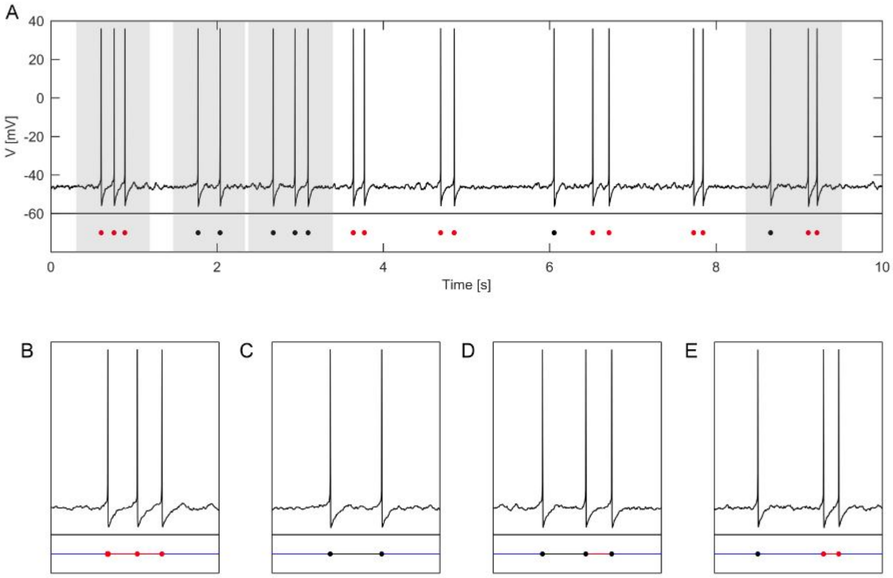

Figure S1: **Calculation of P_C** (A) Example 10s simulation with raster plot of spikes (below), defined as the point at which $V = 0$ and $dV/dt > 0$. Spikes marked in red are classified as clustered spikes, whilst spikes marked in black are not clustered. Clusters are defined as two or more spikes with an ISI of < 250ms and preceeded and followed by quiescent periods of > 300ms. P_C is calculated as the ratio of clustered spikes to total number of spikes; in this example $P_C = 13/20 = 0.65$. (B-E) Shaded grey regions in A. In the raster plots below the traces, red dots are clustered spikes and black dots are non-clustered spikes. Blue lines represent epochs of > 300ms, black lines are epochs of < 300ms but > 250ms, and red lines are epochs of < 250ms. (B) Three clustered spikes. (C) Non-clustered spikes due to an ISI of > 250ms (black line in raster plot) excluding them from being classed as a cluster. (D) Non-clustered spikes since the ISI of the first and second spike is > 250ms and < 300ms. The second and third spike cannot constitute a cluster of two spikes since they are not preceeded by a period of > 300ms. (E) One sporadic spike and a cluster of two spikes. The first spike is classified as sporadic, but because the 2nd and 3rd spikes are now both preceeded and followed by a quiescent period of > 300ms, they constitute a cluster of two spikes.

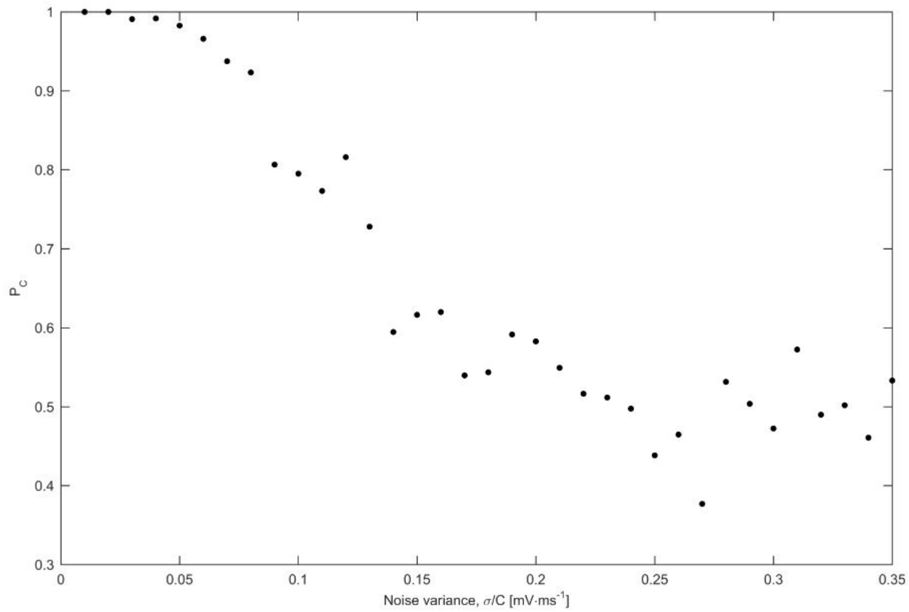

Figure S2: **Effect of noise variance on** P_C Variance of noise σ/C in $\mathrm{mV}\cdot\mathrm{ms}^{-1}$ against P_C for the $(g_\mathrm{h}, g_\mathrm{AHP}) = (2.8, 0.425)$ regime. P_C in the experimental data for the WT animals was found to be 0.69, so noise variance was chosen to be 0.135 $\mathrm{mV}\cdot\mathrm{ms}^{-1}$.

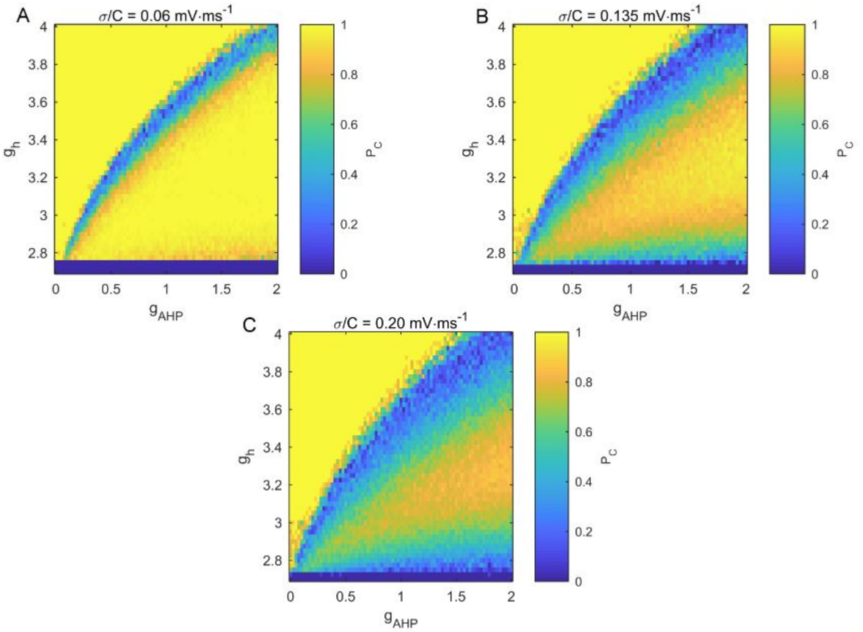

Figure S3: **Effect of noise variance on P_C heatmaps** Two parameter sweeps showing P_C for g_{AHP} vs g_{h}, for different noise values. Whilst the tonic and steady state regimes (in the underlying deterministic system) are largely unaffected by changing noise, in a clustering regime noise has the affect of adding more sporadic spikes and hence scaling P_C. The general pattern of P_C values in the sweep remains the same.

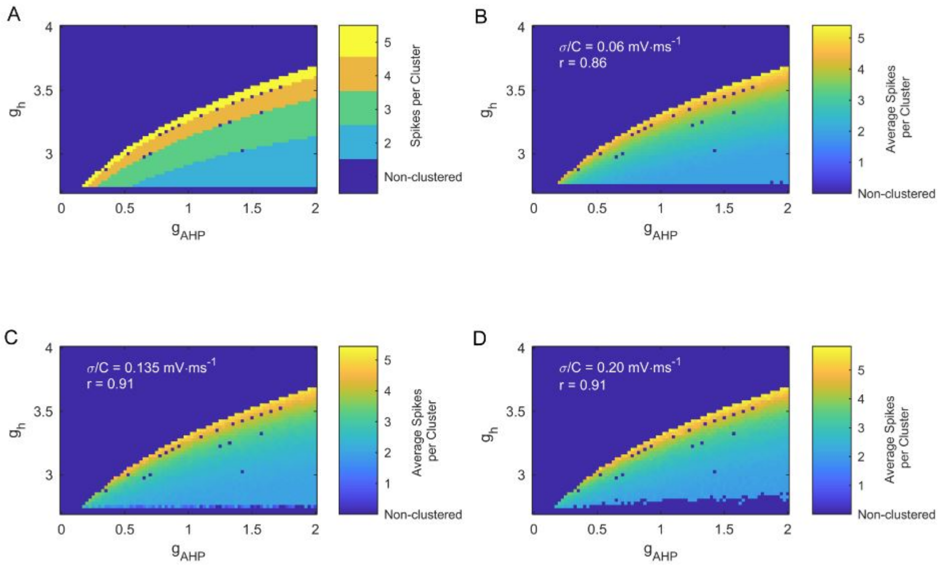

Figure S4: **Spikes per cluster in the stochastic system is correlated with spikes per cluster in the deterministic system.** (A) Spikes per burst in the bursting regimes of the deterministic model. Dark blue regions represent either tonic firing, steady state, or chaotic regimes and are not included in the analysis. (B-D) Mean number of spikes per cluster in the stochastic system with three different levels of noise. Only clustered spikes were included (sporadic spikes ignored) in calculation. Text shows the noise level and correlation of number of spikes per cluster over the parameter sweep between each system and the deterministic system.

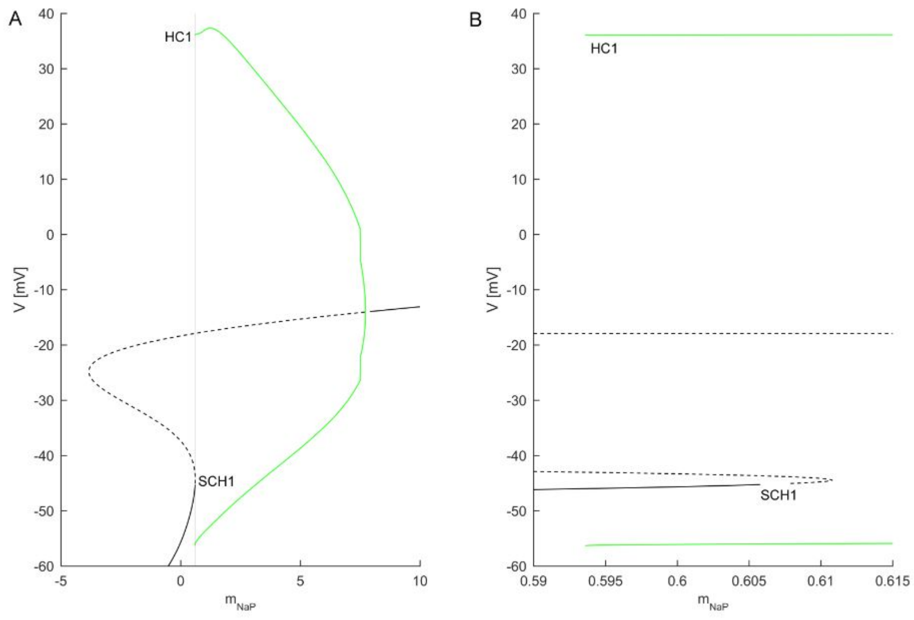

Figure S5: **Bifurcation diagram for $m_{\mathbf{NaP}}$ for a single value of $h_{\mathbf{Kas}}$** (A) Full bifurcation diagram of the fast subsystem whilst $h_{\mathrm{Kas}} = 0.19$. (B) Zoom in on the bistable region of A (shaded in grey) to demonstrate HC1 homoclinic and SCH1 subcritical Hopf bifurcations.

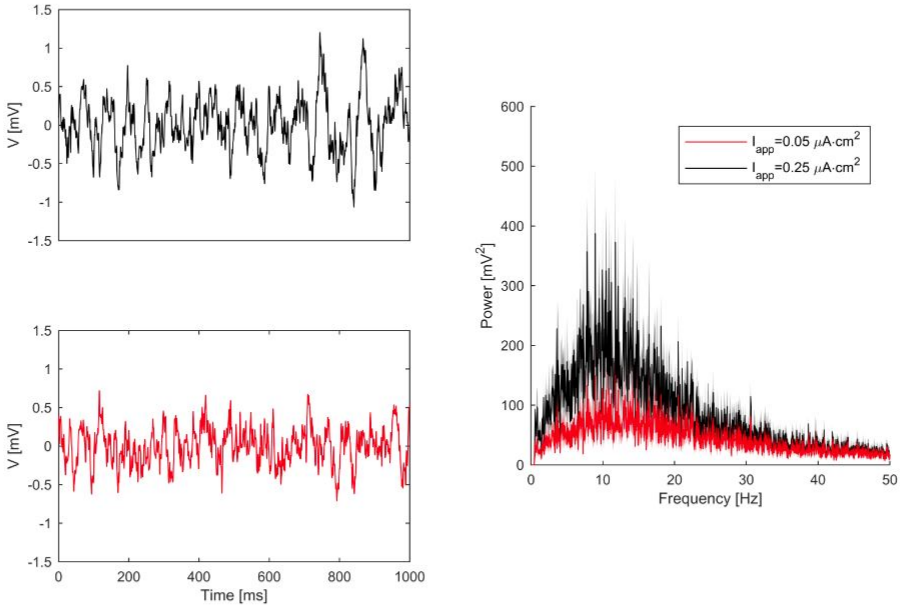

Figure S6: **Subthreshold theta fluctuations in a system with noise variance** $\sigma/C =$ 0.135 $\mathbf{mV{\cdot}ms^{-1}}$ (A) Example membrane potential trace for system close to threshold ($I_{\mathrm{app}} =$ 0.25 μA·cm^{-2}). (B) Example membrane potential trace for system far from threshold ($I_{\mathrm{app}} =$ 0.05 μA·cm^{-2}). (C) Power spectrum for each value of I_{app}, averaged over 10 cells for 20 seconds. Close to threshold, the theta peak is greater, as seen in experiments.

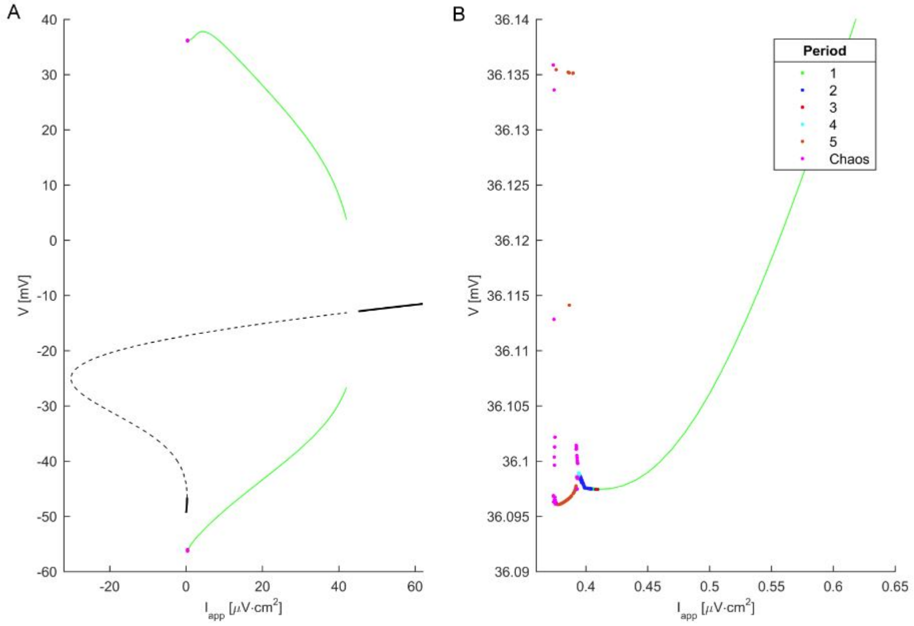

Figure S7: **Bifurcations in I_{app}.** Bifurcations in I_{app} for values of g_{h} and g_{AHP} used in sections 3.2 and 3.3. (A) Full bifurcation diagram for I_{app}. (B) Flip bifurcations cause periodic bursting regimes to occur.

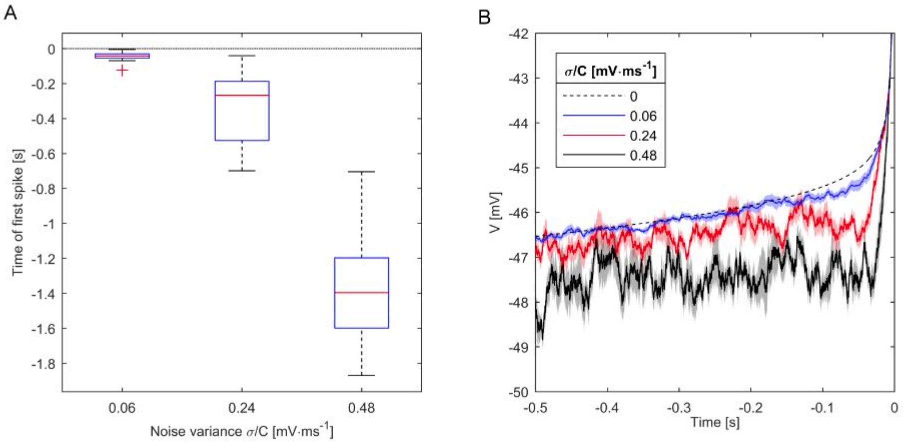

Figure S8: **Response of cell to current ramp** (A) Time of first spike of stochastic neurons (boxplot) during current ramp relative to time of first spike in the deterministic neuron (black dotted line, time = 0). Stochastic neurons fire earlier than the deterministic model in the current ramp experiment due to noise pushing the membrane potential above threshold. The larger the noise, the earlier the neuron fires due to excitability. (B) Each trace represents the mean of 10 simulations with a given noise level. Shaded regions represent standard error over the 10 simulations. Aligning time of first spike (time = 0), the stochastic system tends to exhibit a lower mean mebrane potential prior to the spike, particularly with large noise. This is because the first spike is, on average, earlier in the current ramp (see A), so input current is lower.

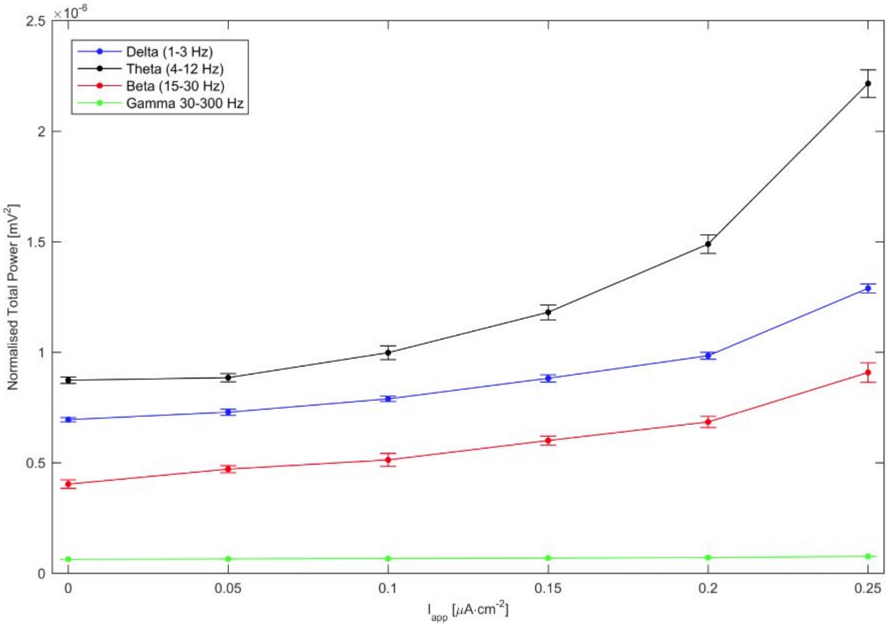

Figure S9: **Effect of $I_{\mathbf{app}}$ on total power in key frequency bands** For the four common electrophysiological frequency bands (see figure legend), total power (normalised by the width of the band) is plotted vs I_{app}. Error bars are standard error over 10 simulations. As action potential threshold (at $I_{\mathrm{app}} = 0.2738\mu\mathrm{A}\cdot\mathrm{cm}^{-2}$) is approached, theta band oscillations become more powerful and become the dominant frequency, reflecting electrophysiological recordings.
